# Supplementary material for: The causal relationship between the human gut microbiota and pyogenic arthritis: a Mendelian randomization study
Source: Front Cell Infect Microbiol. 2024 Nov 26;14:1452480. doi: 10.3389/fcimb.2024.1452480 (PMC11629706; doi:10.3389/fcimb.2024.1452480)
Supplement: Supplementary file 1 [file DataSheet1.zip › mendelian test/ebi-a-GCST90027528.csv_leave_one_out.pdf]

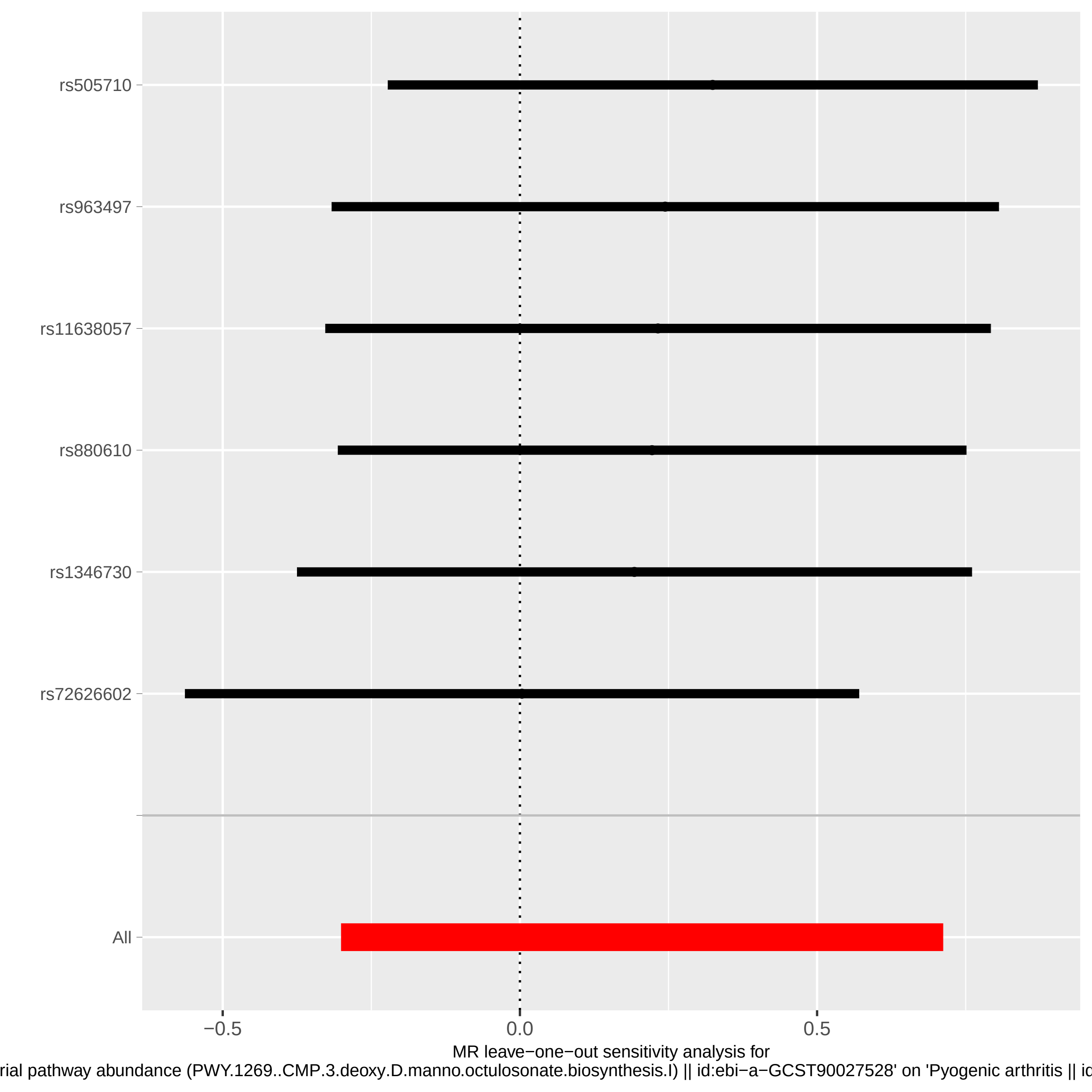

rs505710
rs963497
rs11638057
rs880610
rs1346730
rs72626602
All
−0.5
0.0
0.5
MR leave−one−out sensitivity analysis for
rial pathway abundance (PWY.1269..CMP.3.deoxy.D.manno.octulosonate.biosynthesis.I) || id:ebi−a−GCST90027528' on 'Pyogenic arthritis || id
